# Light-amplification-assisted sum-frequency generation in erbium-doped thin-film lithium niobate optical waveguides

**DOI:** 10.1515/nanoph-2025-0359

**Published:** 2025-11-14

**Authors:** Yan Liu, Zhenzhong Hao, Xiao Wu, Shuting Kang, Rui Ma, Yuchen Zhang, Hongde Liu, Dahuai Zheng, Yongfa Kong, Fang Bo, Guoquan Zhang, Jingjun Xu

**Affiliations:** MOE Key Laboratory of Weak-Light Nonlinear Photonics, TEDA Institute of Applied Physics and School of Physics, 12538 Nankai University, Tianjin, 300457, China

**Keywords:** lithium niobate, optical waveguide, rare earth ions, optical amplification, sum-frequency generation

## Abstract

Erbium-doped thin-film lithium niobate (Er^3+^:TFLN) enables integrated photonic devices through its efficient photoluminescence. However, the fixed transition energies of erbium ions intrinsically restrict emission to the telecommunications C-band (1530–1565 nm), limiting spectral versatility. To transcend this constraint, we engineered periodically poled Er^3+^:TFLN waveguides that concurrently integrate optical amplification and nonlinear frequency conversion. Within this platform, we harnessed erbium ions stimulated emission under 980 nm pumping to achieve net optical gain (0.8 dB) at 1538.2 nm. Simultaneously, we exploited the quasi-phase-matching (QPM) capability of the poled structure to perform sum-frequency generation (SFG) between the 976.0 nm pump and the amplified 1538.2 nm signal. This dual-process yielded visible emission at 597.1 nm with 84 nW output power and a normalized conversion efficiency of 68 % W^−1^ cm^−2^. Critically, this work demonstrates-for the first time in Er^3+^:TFLN-spectral extension beyond the C-band through synergistic pump amplification and nonlinear mixing. Our monolithic architecture establishes a new paradigm for broadband on-chip photonics, enabling applications including multi-wavelength laser sources, quantum entangled photon pair generators, and on-chip biophotonic sensing systems.

## 1 Introduction

Lithium niobate (LiNbO_3_, LN), a multifunctional dielectric crystal, is regarded as an ideal platform for integrated photonic systems owing to its exceptional material properties [1], [2]. These include large second-order nonlinear optical coefficients (*d*_33_ = 27 pm/V), a broad transparency window spanning 0.35–5 μm, and strong electro-optic effects (*r*_33_ = 31 pm/V) [3], [4]. The emergence of thin-film lithium niobate (TFLN) technology has enabled high-performance passive devices such as electro-optic modulators exceeding 100 GHz bandwidth [5], microwave-to-optical transducers [6], and on-chip spectrometers with 0.1 nm resolution [7].

However, the development of fully integrated lithium niobate photonics circuits is constrained by a fundamental material limitation: the intrinsic absence of efficient electroluminescence. This deficiency originates from the indirect bandgap nature of lithium niobate [8], which severely restricts radiative carrier recombination processes. To address this challenge and incorporate active functionalities, rare-earth ion doping-particularly with erbium (Er^3+^) and ytterbium (Yb^3+^) has been employed. This strategy has yielded active TFLN devices including microdisk lasers [9], [10], [11], microring lasers [12], [13], [14], and optical waveguide amplifiers [15], [16], [17].

Nevertheless, the operational wavelengths of erbium doped devices are predominantly confined to the telecommunications C-band due to fixed intra-4f-shell ^4^ *I*_15/2_-^4^ *I*_13/2_ transiton of the dopant ions [18]. Expanding the operational spectrum beyond this range constitutes a critical objective for enhancing the versatility of active lithium niobate photonics. Nonlinear frequency conversion via QPM on TFLN platforms [19], [20], [21] offers a promising pathway to overcome this spectral constraint.

Building on this foundation, we demonstrate a monolithic integration approach by combining an erbium-doped gain medium with a periodically poled structure within a single waveguide-fabricating a periodically poled erbium-doped thin-film lithium niobate (PP–Er^3+^:TFLN) device. Under co-propagating 976.0 nm pump (3 mW) and 1550 nm band signal (0.165 mW) excitation, we observed SFG at 597.1 nm in the visible spectrum. This process achieved a normalized conversion efficiency of 68 % W^−1^ cm^−2^. Notably, under constant pump-signal power product conditions (*P*_p_ × *P*_s_ = constant), the sum-frequency power exhibited marked deviation from conventional nonlinear theory. This unconventional spectral signature confirms the presence of signal amplification through stimulated emission a critical synergy between optical gain and nonlinear processes that facilitates spectral range extension.

## 2 Sample design and fabrication

**Figure 1:**
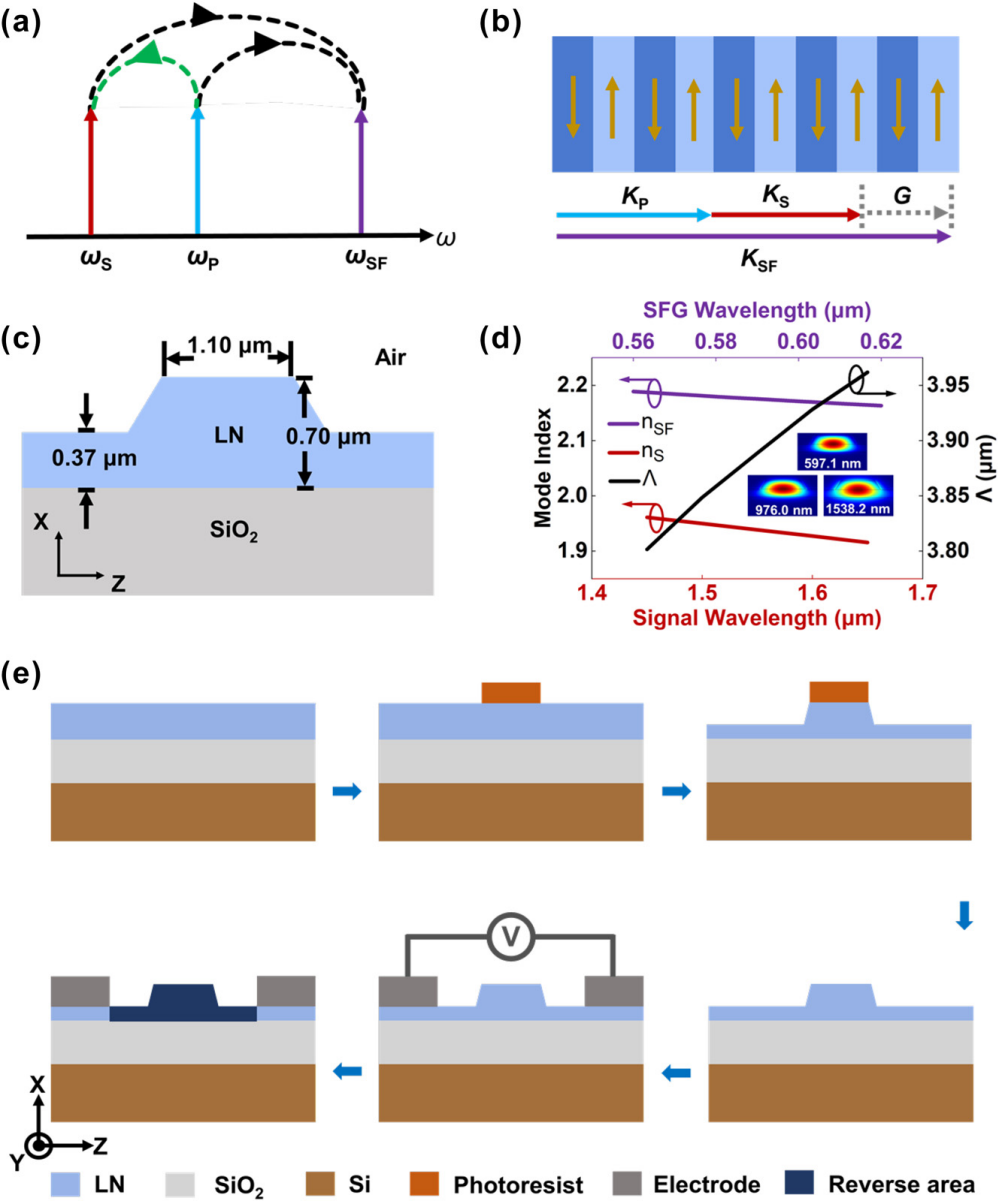
PP-Er^3+^:TFLN waveguide design and fabrication. (a) Schematic diagram of the integrated optical amplification and nonlinear sum-frequency generation process is depicted in the PP-Er^3+^:TFLN waveguide. (b) Schematic of the periodically polarized structure for QPM with the inverted lattice vector of periodically polarized domain structure compensates for the phase mismatch in the SFG process. (c) Schematic diagram of the optical waveguide cross-section, with geometrical parameters labeled. (d) Calculated poling period for QPM as a function of wavelength, determined using the simulated effective refractive indices of the fundamental TE_00_ mode for the waveguide structure shown in (c), the inset shows the simulated TE_00_ mode field distributions for the relevant wavelengths. (e) Flowchart summarizing the fabrication process flow for the PP-Er^3+^:TFLN waveguide.

Erbium ions serve as the optical gain medium in this work, providing amplification within the 1530–1565 nm spectral band through characteristic intra-4f electronic transitions. Although both 980 nm and 1480 nm pumping schemes are viable, we selected the 980 nm pump wavelength due to the higher absorption coefficient of erbium ions at this band [22], thereby maximizing pump absorption efficiency. This design strategy is illustrated in Figure 1(a).

Efficient sum-frequency generation process requires simultaneous satisfaction of two fundamental conditions: the energy conservation (*ω*_S_ + *ω*_P_ = *ω*_SF_), shown in Figure 1(a), and the quasi-phase matching condition (Δ*k* = *k*_SF_ - *k*_S_ - *k*_P_ - G = 0), shown in Figure 1(b). Here, *ω* and *k* denote angular frequency and wave vector, respectively, with subscripts S, P, and SF corresponding to the signal, pump, and sum-frequency. The grating vector is defined as G = 2π/Λ, where Λ denotes the poling period. This relationship is schematically depicted in Figure 1(b). To determine the required QPM period, we first computed the effective refractive indices at the signal, pump, and sum-frequency wavelengths. Using the finite element method (FEM), we simulated the fundamental transverse-electric (TE_00_) mode field distribution based on the waveguide cross-section geometry shown in Figure 1(c). Subsequently, the target QPM period was calculated using the QPM period formula [23]. The calculated dependence of Λ on wavelength is presented in Figure 1(d). For our target wavelengths-the signal at 1538.2 nm, pump at 976.0 nm, and SFG at 597.1 nm-the required QPM period was calculated to be 3.85 μm.

The device fabrication utilized an X-cut Er^3+^:TFLN with the following structure: a 700 nm-thick Er^3+^:TFLN layer (doping concentration: 0.1 mol%), a 2 μm-thick SiO_2_ buffer layer, and a 500 μm silicon substrate. The fabrication process flow for the PP-Er^3+^:TFLN waveguide is outlined in Figure 1(e), comprises the following key steps: First, hydrogen silsesquioxane (HSQ) electron beam resist was spin-coated onto the Er^3+^:TFLN. Subsequently, the waveguide structure was defined via electron beam lithography (EBL) [24]. Following lithography, the pattern was transferred into the Er^3+^:TFLN with inductively coupled plasma reactive ion etching (ICP-RIE). After etching, residual HSQ was stripped using a buffered hydrofluoric acid (HF). Next, comb-shaped, 250 nm-thick aluminum electrodes (see Section 1 of the Supplementary Material) were fabricated: this involved patterning the electrode design by EBL, followed by electron beam evaporation of aluminum and lift-off processing. Finally, the ferroelectric domain structure was formed by applying an electric field across the electrodes (see Section 1 of the Supplementary Material).

**Figure 2:**
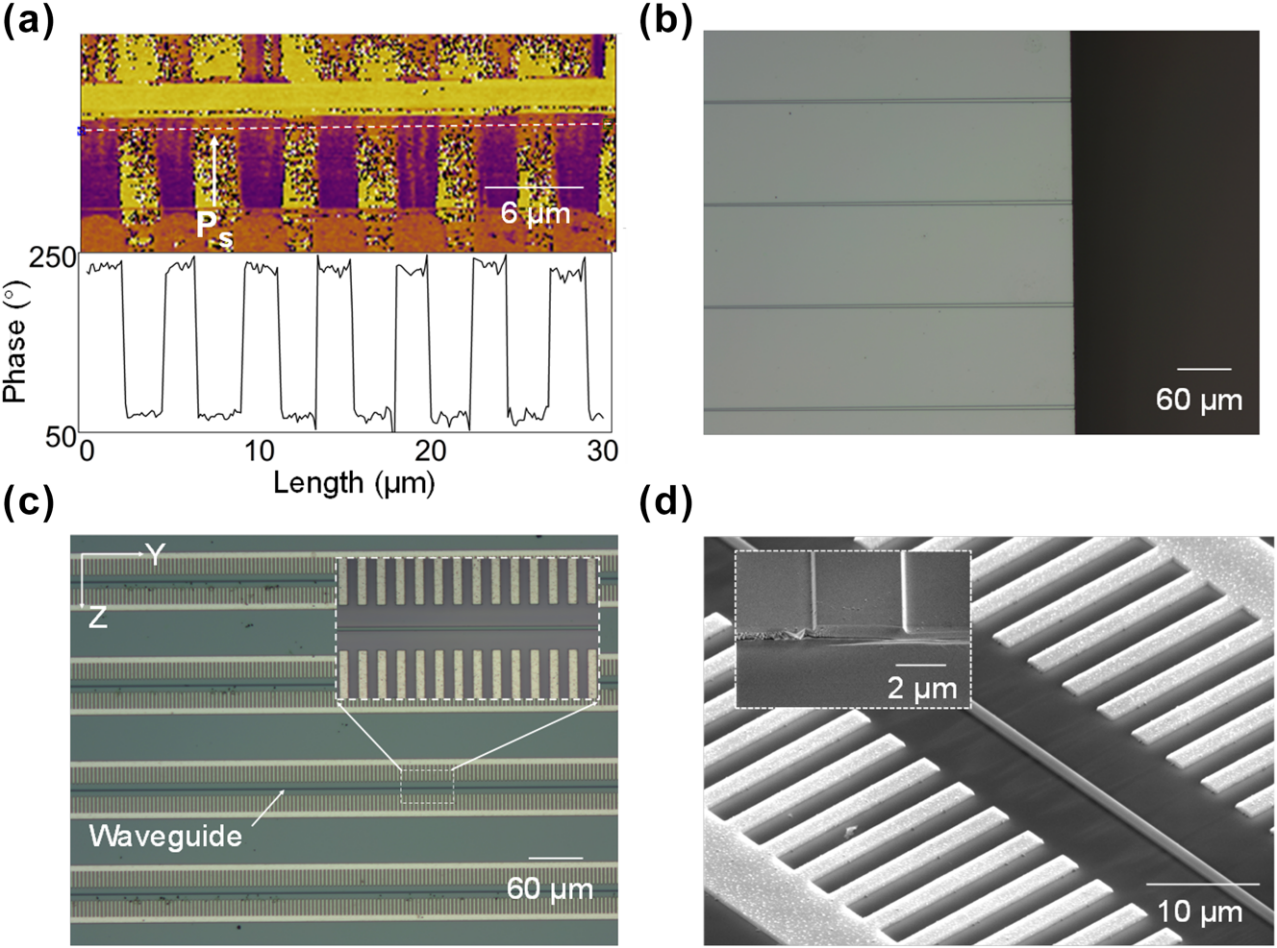
Structural characterization of the PP-Er^3+^:TFLN waveguide. (a) PFM phase image of periodically poled domain structures. White arrows indicate the orientation of spontaneous polarization within individual domains. The phase profile along the white dashed line demonstrates the 180° phase shift between adjacent domains, confirming antiparallel polarization alignment. (b) Optical micrograph of the waveguide facet region. (c) Optical micrograph of the PP-Er^3+^:TFLN waveguide array with aluminium comb electrodes used for electric-field poling. Inset: magnified view of the region within the dashed white rectangle. (d) SEM image of the waveguide ridge and adjacent electrode structure. Inset: magnified view of the waveguide facet.

Following waveguide fabrication, domain structures and device morphology were systematically characterized using piezoresponse force microscopy (PFM), optical microscopy, and scanning electron microscopy (SEM). As shown in the PFM phase image of Figure 2(a), periodically poled ferroelectric domain structures are clearly visible in the substrate regions flanking the optical waveguide ridge. The distinct 180° phase contrast between adjacent domains confirms successful domain inversion to a finite depth beneath the ridge. Notably, however, no domain inversion is detected on the top surface of the waveguide ridge. The resulting incomplete polarization reduces the efficiency of nonlinear frequency conversion. Quantitative analysis further indicates a duty cycle of approximately 45 %, defined as the ratio of the inverted domain width to the poling period, which is consistent with the design target.

The total length of the waveguide is 6 mm, with a periodically poled region measuring 5 mm in length. Regarding optical mode coupling, Figure 2(b) displays an optical micrograph near the waveguide facet. To enhance fiber-to-waveguide coupling efficiency, a linear taper structure was implemented near the input/output end faces. This taper adiabatically expands the waveguide width from 1.1 μm to 3.0 μm along its length, optimizing spatial overlap between the guided modes and the optical fiber mode field [25]. Furthermore, Figure 2(c) shows an optical micrograph of the periodically poled region, featuring an array of fabricated PP-Er^3+^:TFLN waveguides. Notably, the comb electrodes used for electric field poling remain intact post-poling, exhibiting no damage, confirming the applied voltage remained safely below LN breakdown threshold. The inset provides a magnified view a section of a single waveguide. Figure 2(d) shows the SEM images of the optical waveguide and polarized electrode, in which the three-dimensional structure of the optical waveguide and the electrode can be observed more clearly, and the periodically polarized domain structure can also be faintly seen under close observation, and the inset shows the facet of the PP-Er^3+^:TFLN waveguide.

## 3 SFG and optical amplification

**Figure 3:**
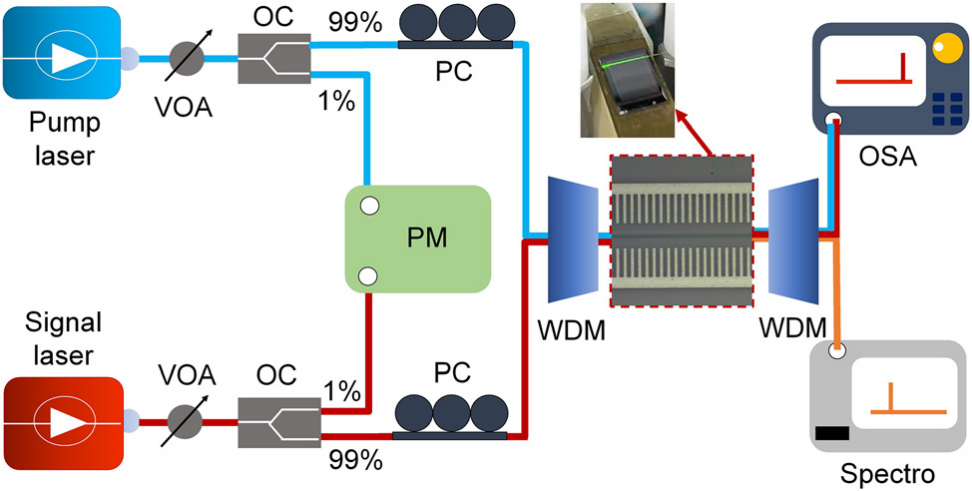
Schematic diagram of the experimental setup. VOA, variable optical attenuator; OC, optical coupler; PC, polarization controller; PM, power meter; WDM, wavelength-division multiplexer; OSA, optical spectrum analyzer; Spectro, grating spectrometer. The inset (indicated by the red arrow) shows green fluorescence generated within the PP-Er^3+^:TFLN waveguide. Optical paths are color-coded: blue (976.0 nm pump), red (1538.2 nm signal), and orange (597.1 nm SFG output).

To characterize the SFG and optical amplification properties of PP-Er^3+^:TFLN waveguides, we implemented a dual-wavelength pump-probe system (Figure 3) comprising a 980 nm-band continuous-wave pump laser and a tunable 1550 nm-band signal laser. Both beams were conditioned through variable optical attenuator (VOA), 99:1 optical coupler (OC), and polarization controller (PC), then combined via a wavelength-division multiplexer (WDM) for lensed-fiber coupling into the waveguide. Real-time input power monitoring utilized a 1 % tap port from an optical coupler (OC) connected to a power meter. Output light was collected by a lensed fiber and spectrally separated into two detection paths: C-band signals were routed to an optical spectrum analyzer (OSA) for amplification characterization, while visible wavelengths were measured by a grating spectrometer. All input and output fiber ends were mounted on a vibration-isolated optical platform throughout the experiment. Prior to formal data acquisition, an extended pre-alignment procedure was carried out to optimize coupling stability. Additionally, all connections were thoroughly cleaned and firmly secured. Strong fluorescence observed under 976.0 nm pumping (Figure 3 inset) confirmed efficient erbium-ion absorption. For rigorous SFG efficiency quantification, we characterized the optical waveguide transmission loss and the coupling loss between the fiber-waveguide at relevant wavelengths.

Utilizing the Fabry–Perot cavity transmission spectrum model [26], we fitted the optical waveguide transmission data to quantify propagation losses near key wavelengths: 2.8 dB/cm at 1538.2 nm, 3.06 dB/cm at 976.0 nm, and 3.95 dB/cm at 633.0 nm. For the 597.1 nm wavelength, we inferred transmission loss by fitting spectra acquired with a 633.0 nm source, leveraging the comparable optical properties between these visible wavelengths [27]. The dominant loss mechanisms comprise waveguide sidewall scattering and erbium-ion absorption [16]. Considering symmetric waveguide end-facets, we derived fiber-to-chip coupling efficiencies accounting for insertion loss between dual fiber lenses and on-chip transmission loss [20]. The calculated values are 0.075 (976.0 nm), 0.2 (1538.2 nm), and 0.12 (633.0 nm). Finite-element simulations verified near-identical end-facet coupling characteristics at 633.0 nm and 597.1 nm. Consequently, we characterized lensed-fiber-to-waveguide coupling loss for the 597.1 nm SFG signal using the 633.0 nm laser source [28].

**Figure 4:**
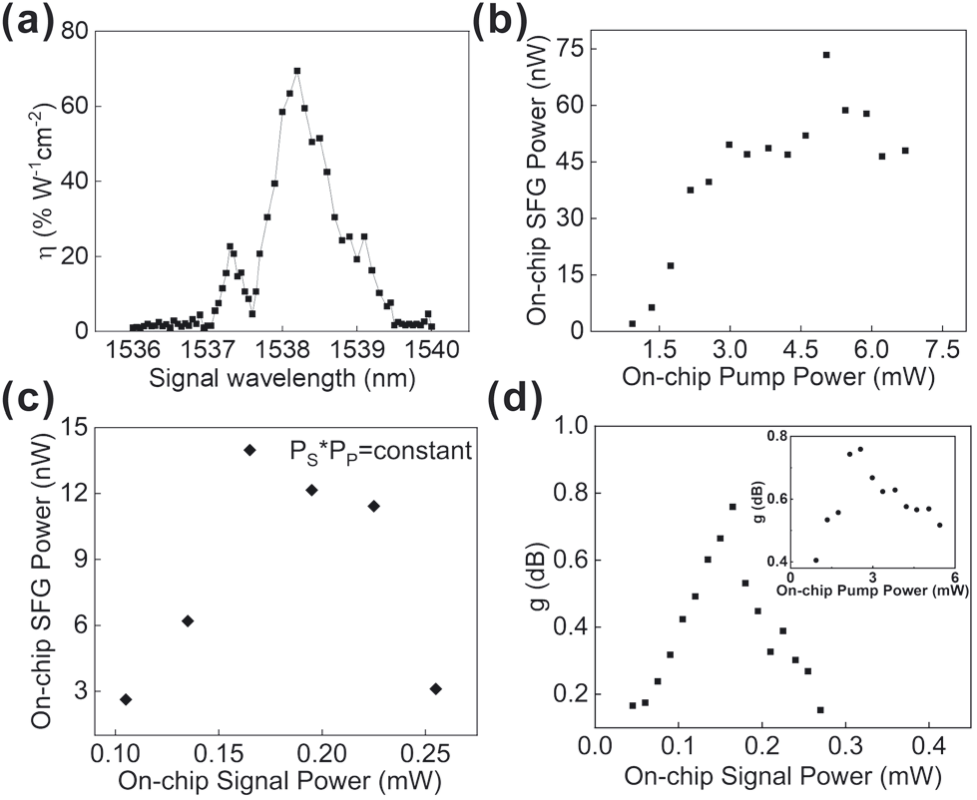
Experimental characterization of SFG and optical amplification. (a) *η* versus signal wavelength (*λ*_S_) at fixed pump wavelength (*λ*_P_ = 976.0 nm), pump power (*P*_P_ = 3 mW), and signal power (*P*_S_ = 0.165 mW). (b) SFG output power versus pump power at *λ*_P_ = 976.0 nm, *λ*_S_ = 1538.2 nm, and *P*_S_ = 0.5 mW. (c) SFG power versus product of pump and signal power at *λ*_P_ = 976.0 nm and *λ*_S_ = 1538.2 nm. (d) C-band gain versus signal power at fixed *P*_P_ = 2.6 mW, the inset C-band gain versus pump power at fixed *P*_S_ = 0.165 mW.

In the optical amplification and nonlinear frequency conversion experiments, we systematically investigated conversion efficiency of the SFG by varying the power and wavelength of the pump and signal, while monitoring the power of the SFG output. For the SFG process, the normalized conversion efficiency is defined as *η* = *P*_SF_/(*P*_S_·*P*_P_·*L* ^2^) [23], where *P*_P_, *P*_S_, and *P*_SF_ denote the power of pump, signal, and SFG, respectively, and L indicates the length of the PP-Er^3+^:TFLN waveguide. As depicted in Figure 4(a), we measured the normalized conversion efficiency as a function of signal wavelength under fixed pump conditions. With the pump wavelength stabilized and pump power maintained at 3 mW, we tuned the signal wavelength while keeping signal power constant at 0.165 mW. From Figure 4(a), we can see that the SFG occurs with signal wavelength at 1538.2 nm, exhibiting a peak normalized conversion efficiency of 68 % W^−1^ cm^−2^ with a 1.1 nm full-width-at-half-maximum (FWHM). This experimental peak efficiency is substantially lower than the theoretical prediction of 5,200 % W^−1^ cm^−2^. We attribute this discrepancy principally to three factors: incomplete poling domain penetration through the waveguide cross-section [29], which reduces the effective nonlinear interaction length; deviation from the ideal poling duty cycle [30], and film thickness non-uniformity [31]. Figure 4(b) shows the SFG output power as a function of pump power under fixed signal conditions (*λ*_s_ = 1538.2 nm, *P*_s_ = 0.5 mW) and pump wavelength (*λ*_p_ = 976.0 nm). As the pump power increases, thermal effects become significant, destabilizing the waveguide-fiber coupling and resulting in a reduction of the measured sum-frequency power. Upon re-optimizing the coupling condition, the sum-frequency power recovers and increases within the pump power range of 4.5–6 mW. With further elevation of pump power, the rising temperature perturbs the initial phase-matching conditions, leading to a decline in sum-frequency power and eventual saturation.

To investigate the influence of C-band signal gain on SFG in the PP-Er^3+^:TFLN waveguide, we maintained a constant product of pump and signal power while varying individual power and monitoring SFG output. Figure 4(c) shows the variation in sum-frequency power for six different combinations of pump and signal powers, with the product of their powers held constant at 0.42 mW^2^. The maximum sum-frequency generation power occurs at a pump power of 2.5 mW and a signal power of 0.165 mW. Theoretically, if the power of the signal is independent of the pump intensity (i.e., without erbium-ion-mediated amplification), the SFG power should theoretically remain constant. The measured deviation demonstrates that the signal amplification within the PP-Er^3+^:TFLN waveguide influences the SFG efficiency. Based on further investigations at a fixed pump power of 2.5 mW, the signal gain remains low under weak input signal power conditions (Figure 4(d)). This behavior can be explained by the fact that at low signal power, a significant portion of the signal light is efficiently converted into sum-frequency through the SFG process. As a result, the linear gain mechanism remains largely inactive due to insufficient photon density to stimulate substantial emission, leading to low signal power and minimal net gain. When the signal power exceeds 0.165 mW, gain compression becomes evident in the C-band. This behavior can be attributed to a dual mechanism. Firstly, at higher optical power levels, excited-state absorption in erbium ions becomes significantly enhanced, competing with and potentially surpassing the stimulated emission process, which substantially reduces the net optical gain. Secondly, the amplification of the signal light can no longer fully compensate for the energy depletion resulting from sum-frequency generation. The inset of Figure 4(d) shows C-band gain versus pump power at the wavelength (1538.2 nm) and the power of the signal is 0.165 mW. Rapid gain increase at low pump powers characterizes the small-signal regime, while net gain reduction occurs beyond 2.5 mW. This saturation stems from pump power consumption through upconversion fluorescence in the erbium doped film, compounded by significant pump depletion from its participation in the SFG process which diverts power from signal amplification. The relatively low gain reported here stems from a combination of intrinsic loss mechanisms such as upconversion, uneven pump depletion along the waveguide, and fabrication imperfections that limit the interaction length and detune the phase-matching condition from the 1532 nm peak. Future enhancements will focus on employing inhomogeneous doping, bidirectional pumping, and resonant cavities to overcome these limitations.

A set of coupled-mode equations was established to model the evolution of the signal and SFG in a PP-Er^3+^:TFLN waveguide, incorporating both optical amplification and sum-frequency generation processes. The theoretical results obtained from this model show good agreement with experimental data (see Section 2 of the Supplementary Material), confirming that erbium doping is linked to the occurrence of nonlinear effects such as signal saturation.

## 4 Conclusions

In this paper, we demonstrate PP-Er^3+^:TFLN waveguide devices fabricated via electron beam lithography, inductively coupled plasma reactive ion etching and external electric field poling. This platform achieves the first synergistic integration of optical amplification and QPM nonlinear frequency conversion: a 980-nm pump simultaneously excites erbium-ions gain in the C-band and generates SFG with amplified signal under QPM conditions. For 976.0 nm pump (3 mW) and 1538.2 nm signal (0.165 mW) inputs, we observe 597.1 nm SFG with 68 % W^−1^cm^−2^ normalized conversion efficiency. Power-dependent characterization confirms concurrent nonlinear frequency conversion and erbium-ion gain within the waveguide. Compared to conventional discrete systems (erbium-ions doped fiber amplifiers + PPLN modules), this integrated device reduces component count by 50 %, enabling compact implementations for self-amplified microwave photonics and quantum frequency conversion applications.

## Supplementary Material

Supplementary Material Details
