# Supplementary material for: Light-amplification-assisted sum-frequency generation in erbium-doped thin-film lithium niobate optical waveguides
Source: Nanophotonics. 2025 Nov 14;14(26):4713–9. doi: 10.1515/nanoph-2025-0359 (PMC12714036; doi:10.1515/nanoph-2025-0359)
Supplement: Supplementary file 1 — Supplementary Material Details [file j_nanoph-2025-0359_suppl_001.pdf]

Light-amplification-assisted sum-frequency generation in erbium-doped thin-film lithium niobate optical waveguides: supplemental document

Yan Liu[1], Zhenzhong Hao[1*], Xiao Wu[1], Shuting Kang[1], Rui Ma[1], Yuchen Zhang[1], Hongde Liu[1], Dahuai Zheng[1], Yongfa Kong[1], Fang Bo[1*], Guoquan Zhang[1], and Jingjun Xu[1]

[1]MOE Key Laboratory of Weak-Light Nonlinear Photonics, TEDA Institute of Applied Physics and School of Physics, Nankai University, Tianjin 300457, China

1 Electrode and polarization pulse

As illustrated in Figure S1(a), the electrode structure consists of a 40 μm × 40 μm pad, a 10 μm × 5 mm connecting electrode, and a toothed electrode with a width of 1.1 μm and a length of 20 μm. The gap between the positive and negative electrode teeth was set to 10 μm. A typical poling voltage waveform is presented in Figure S1(b), which comprises three pulses. The ferroelectric domain structure was induced by electric field poling under the following conditions: an ambient environment of ~20 °C and ~50% relative humidity, and poling parameters of 190 V, 20 ms pulse width, 1 Hz frequency, and 20 pulses.

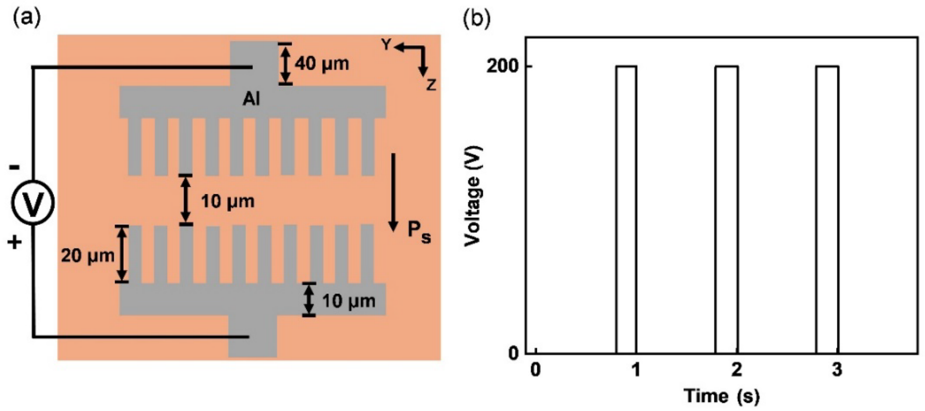

Figure S1: (a) Schematic of the domain inversion electrode structure. (b) Applied poling voltage waveform, consisting of three pulses.

2 Coupled-wave equations for simultaneous sum-frequency generation and gain

Equation (S1) describes the coupled-mode model governing the interplay between the nonlinear and gain processes. In our experiment, the total length of the waveguide is 6 mm, with a 5 mm periodically poled region.

$$\begin{aligned}
\frac{dA_p}{dz} &= i\omega_p \kappa_{sf} A_s^* A_{sf} e^{-i\Delta kz} + \frac{\alpha_{abs}}{2} A_p \\
\frac{dA_s}{dz} &= i\omega_s \kappa_{sf} A_p^* A_{sf} e^{-i\Delta kz} + \frac{g_s}{2} A_s \\
\frac{dA_{sf}}{dz} &= i\omega_{sf} \kappa_{sf} A_p A_s e^{-i\Delta kz}
\end{aligned} \tag{S1}$$

In Equation (S1), the subscript p,s,sf denote the pump, signal, sum-frequency waves, respectively. The other parameters are defined as follows: $A_{p,s,sf}$ represents the complex amplitude; $\omega_{p,s,sf}$ denotes the angular frequency; $\kappa_{p,s,sf}$ is the nonlinear coupling coefficient; α_{abs} corresponds to the absorption coefficient of the pump, $\alpha_{abs} = (N_3 - N_1)\sigma_p$; N_1 and N_3 represent the number of particles in levels 1 and 3, with N_3 is close to 0; σ_p stands for the absorption cross-sections of the pump; g_s is the gain coefficient of the signal, $g_s = (N_2 - N_1)\sigma_s$, $N_2 - N_1 = \frac{\phi_p \sigma_p - \Gamma_{21}}{\Gamma_{21} + 2\phi_s \sigma_s + \phi_p \sigma_p} N$, N_2 refers to the number of particles in levels 2, N indicates the total doping concentration of erbium ions; σ_s is the absorption cross-sections of the signal, $\phi_s = \frac{I_s}{h\nu_s}$, $\phi_p = \frac{I_p}{h\nu_p}$, $\phi_{p,s}$ and $I_{p,s}$ denote the photon flux and intensity, respectively; $I = \frac{P\Gamma}{A_{eff}}$, P represents the power, Γ is the overlap factor, A_{eff} indicates the effective cross-section of the erbium ion distribution, Γ_{21} is the probability of relaxation from level 2 to level 1, $\Gamma_{21} = 1/\tau_2$, τ_2 stands for the lifetime of energy level 2.

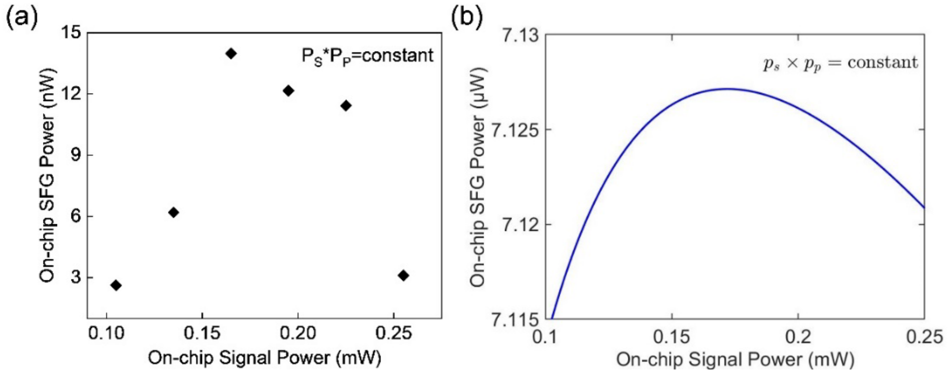

Figure S2: The dependence of the sum-frequency power on the product of the pump and signal powers. (a) Experimental data; (b) Numerical simulations.

We leveraged Equation (S1) to simulate the variation in sum-frequency power under the condition of a constant product of pump and signal power. The numerical values of the simulation-related

parameters are as follows. $N = 1.5e^{19}$ /cm^3 , $\tau_2 = 2.7\ ms$, $\sigma_p = 5.5e^{-21}$ cm^2 , $\sigma_s = 2.4e^{-20}$ cm^2 , $\Gamma = 1$, $A_{eff} = 0.5\ \mu$m^2 , the remaining parameters are described in the article. Figure S1(a) presents the experimental results, while Figure S1(b) displays the corresponding simulation results. Both the experimental and simulation results indicate that, due to the involvement of the gain process, the sum-frequency power varies with increasing signal power.

The evolution of signal gain versus input signal power at a fixed pump power was also simulated using Equation (S1). Both experimental (Figure S3(a)) and simulation (Figure S3(b)) results indicate that the gain is low at low signal power. As the signal power increases, the gain initially rises and then decreases.

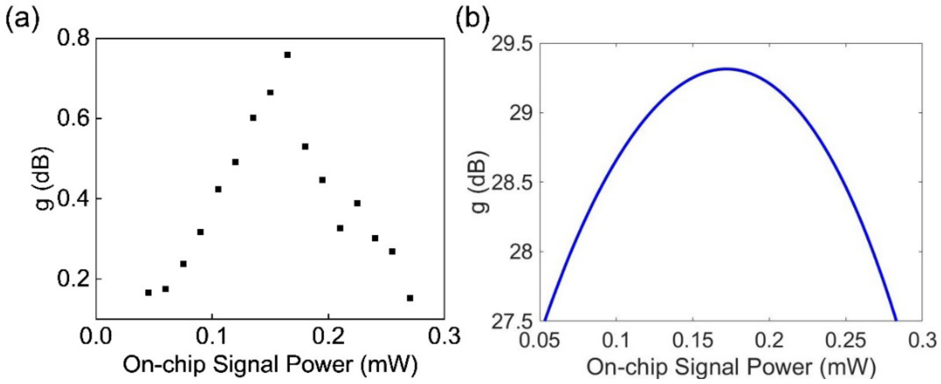

Figure S3: (a) The experimental and (b) simulation results for the signal gain versus input power. Both datasets demonstrate the gain initially rises and then falls with increasing power.
